# Optimization of Prescription Drug Monitoring Program to Overcome Opioid Epidemic in West Virginia

**DOI:** 10.7759/cureus.22434

**Published:** 2022-02-21

**Authors:** Ala-Eddin Yassin Al-Astal, Komal Sodhi, Hari Vishal Lakhani

**Affiliations:** 1 Biomedical Sciences, Marshall University Joan C. Edwards School of Medicine, Huntington, USA; 2 Surgery and Biomedical Sciences, Marshall University Joan C. Edwards School of Medicine, Huntington, USA; 3 Cardiology, Surgery and Biomedical Sciences, Marshall University Joan C. Edwards School of Medicine, Huntington, USA

**Keywords:** utilization, implementation, functional limitations, perceived benefits, prescription drug monitoring program

## Abstract

The development of the Prescription Drug Monitoring Program (PDMP) led to an innovation in the healthcare organization system (HCOs). The PDMP system has been utilized in different states at various organizational levels in an effort to achieve improved health outcomes, reduce the number of prescription drug overdoses, and lighten the economic burden that follows. However, during the implementation of PDMP, there were several barriers and limitations that were discovered. Those barriers impeded the process of utilization of PDMP, such as the complex user interface and lack of training for healthcare providers. The purpose of this paper was to examine the advances and limitations in the utilization and implementation of PDMP in the US healthcare industry and develop strategies for effective use of PDMP in West Virginia.

The qualitative part of this paper was a literature review. The paper referred to several peer-reviewed studies and research articles from several reliable resources, which were reached by databases or Google Scholar. A total of 44 articles were reviewed for this study.

The implementation of the PDMP was influenced by benefits and barriers. This article reviewed several studies in general that demonstrated positive outcomes from the implementation of PDMP, including a reduced number of prescription drug overdoses, coordinated care for patients, and improved health outcomes. However, the barriers and limitations were not neglected, which mainly include integration of PDMP into the electronic health record (EHR) system, lack of training for the providers, and lack of basic standards for the use of PDMP.

Although the new health reforms encouraged the adaption of PDMP among providers, data reporting and data interpretation still remain major concerns for assessing the health outcomes of PDMP implementation.

## Introduction and background

Rules and regulations, new healthcare reforms, and the ever-growing healthcare industry in the United States, highly affect the overall quality of care and health outcomes. Statistically, the overall US spending on healthcare has exceeded $4.1 trillion or 19.7% of the total Gross Domestic Product (GDP), accounting for greater than $12,530 per person, in 2020, which shows an approximate 9.7% increase, as compared to just 4.3% increase seen in 2019 [1]. These figures simply make the US the topmost country with the highest healthcare spending among the top Organisation for Economic Co-operation and Development (OECD) nations [2]. One of the major factors that attribute to the cost of overall healthcare spending is the increasing abuse of prescription drugs. As a part of chronic pain management treatment and several critical conditions, physicians often prescribe narcotics and opioid medications that are later abused by patients [3]. The number of hospital visits related to prescription drug overdoses and misuse has almost doubled from 214 visits/100,000 people in 2004 to 458 visits/100,000 people in 2011 [4]. Similarly, there has been a significant increase in deaths by drug overdose, with the age-adjusted rate of drug overdose deaths increasing from 6.1/100,000 population in 1999 to 21.7 in 2017 [5]. This breakdown of this trend of deaths from drug overdose shows that the rate increased on average by 10% per year from 1999 to 2006, an increase of 2% per year from 2006 to 2013, and an increase of 14% per year from 2013 through 2016 [5]. Multiple studies have shown that there has been an increasing number of deaths from a drug overdose in West Virginia and the state has been noted to be facing a drug overdose epidemic over the last couple of years [6,7]. West Virginia has been noted as one of the top 10 states with the highest age-adjusted drug overdose death rates at 51.5/100,000 standard population [5]. In an effort to control the dispensing of prescription drugs and the resulting addiction, there has been a statewide implementation of prescription drug monitoring programs (PDMP). PDMPs typically collect and store the information for the quantity and the type of prescription drug dispensed to the patient, which can be further utilized by the health care providers, pharmacies, other prescribers, and law enforcement agencies to regulate its use [8]. The use of PDMP has the prospect of potentially limiting the increasing number of overdose cases every day. Although 49 states have taken the initiative for the implementation of the PDMP model, the utilization is still limited by the prescribers [9].

Even though PDMP has the potential to reduce the number of incidences of prescription drug overdoses, it can also offer a possible solution to the increasing healthcare costs due to such cases. There have been multiple studies that provide significant data on the negative impact of prescription opioids on health and their resulting addiction. However, only a limited number of studies have shown concerns over the economic burden that it brings [3,10,11]. According to a recent study, the overall healthcare cost per patient varied from $15,884 to $18,388 among patients who were diagnosed with prescription drug-related conditions, as opposed to $1,830 to $2,210 among a similar group of patients who had no past or current medical history of prescription drugs [12]. Another similar study depicted a threefold increase in the overall US healthcare spending on prescription drug cases, from $2.3 billion in 1999 to $7.4 billion in 2012 [13].

Considering the health benefits over risk ratio and the impact of prescription drugs on healthcare spending, it is important to completely implement the PDMP model in the healthcare industry, which includes hospitals, outpatient clinics, and pharmacies, to control and regulate the distribution of prescription drugs. Implementation of the PDMP model will allow prescribers to access the patient’s database and review the patient's history about the quantity, dose, and time interval of the prescription refills. This review will assess the importance of PDMP in reducing healthcare costs by implementing it in an effective way in order to improve prescribing outcomes and reduce the number of fatalities. PDMP will also result in controlling the prescription drug overdose epidemic in the state of West Virginia and benefit its population by controlling their healthcare costs.

## Review

Methods

A literature review was completed by a systematic search of PubMed, Source Premier Database, Academic Search Premier, Academic Business, LexisNexis, and Google Scholar. It was considered valuable for all the information regarding electronic medical records used in hospitals. This research was limited to sources only available in the English language.

The databases were using the key terms "prescription drug monitoring program" or "costs," "utilization" or "implementation," and "hospital" and "in the US" The search was limited to articles published from 2005 to 2021 to keep the search current. Those articles are considered suitable under the following definitions: prescription drug monitoring programs digitally store controlled substance dispensing information and make that data accessible to prescribers, pharmacies, and law enforcement officials. Relevant articles were selected after a review of abstracts was performed. From a total of 89 references found, 44 were chosen for this research.

This literature review focused on three topics for PDMP use in US hospitals: benefits, limitations, and cost-effectiveness. The implementation of PDMP use in hospitals around benefits was compared through sources to commonalities, while benefits of significance were also included regardless of concurrence with other sources.

Results

Positively Utilizing PDMP in US Hospitals by Prescribers

Healthcare providers are utilizing the PDMP in a variety of ways, which are proving to be beneficial for the healthcare system. A study showed that prescribers have been utilizing the PDMP for administrative purposes, including the verification of the prescription history and current prescription refills [14]. The use of PDMP is often reported in cases where the patient is tested positive for urine drug screening and when suspicious behavior is noted in the physical exam. Another aspect of utilizing PDMP has also been noted in the patients’ coordination of care with another clinician [14,15]. The PDMP database includes the name of the prescribing physician and provides detailed information on the type of drug prescribed, including the quantity and doses that were prescribed. The participants of PDMP can easily contact other physicians to discuss the treatments, diagnosis, and prescribing plans for the patient, to obtain detailed information, if the prescribing practice is deemed inappropriate [16,17]. This practice can eliminate the over-prescribing of such opioids and analgesics, which can be easily abused by the patient and can possibly lead to addiction. A recent study reported that the use of PDMP and the information obtained from it by the healthcare provider influenced them to limit the prescription of controlled medications to the patient [18]. Furthermore, the use of PDMP also led physicians to increase communication with the patients, provide education and counseling, and increase referrals and care coordination for the patients [18].

Additionally, studies have shown that PDMP is even utilized in pharmacy practices to avoid overprescription and regulate prescription opioids [19,20]. The PDMP model is integrated into the pharmacy systems, and they are one of the important participants in handling prescription information. The pharmacists would often contact the prescribers to verify the PDMP data and evaluate the information proactively to inhibit inappropriate prescription patterns [21]. Such practice can even avoid false prescriptions for controlled medications being generated and reported to the appropriate agencies. Apart from that, one of the important aspects of controlling this prescription opioid abuse is patient participation and cooperation. Some of the PDMP participants reported that they often communicate with patients and share their PDMP reports in an effort to achieve cooperation with them [14]. This communication allows prescribers to educate patients about the potential addiction of the prescribed medication and further provide awareness about medication safety. If the patient is already prescribed opioids by other prescribers and the patient shows signs of addiction, it is important to assess and evaluate those signs and take action to prevent further worsening of conditions. This can be achieved by recommending patients to participate in drug rehabilitation programs in their early stages and immediately stop all the potential addictive medications that have been prescribed by choosing alternate medication options [22,23].

Assessing Limitations of PDMP Design and Implementation

Multiple variations have been noted in the PDMP design across different states that have implemented the PDMP model [24]. These variations are often the result of the different technical and political backgrounds of each state, which further complicates the use of PDMP by the prescribers. Participation by the prescribers is often limited, highly depending on the health care institution and the requirements of the specific states [25]. A recent study showed that healthcare providers perceived the use of PDMP data to have low value in terms of its potential usefulness and caused administrative burdens as well as demonstrated concerns over the operability of the PDMP system [26]. The drugs that are monitored under PDMP vary, with almost 45 states allowing regulation of schedule II-IV drugs, while 34 states allow regulation of schedule V drugs [27]. These regulations have been extended in the state of Oregon, where Schedule VI substances are monitored as well, which resulted in an almost 38% reduction in prescription drug overdose cases [28]. The current PDMP model in West Virginia is among the only three states that automatically send their prescription drug reports, for each individual patient, to the law enforcement agencies, licensing boards, pharmacies, and physicians [27].

However, the variation in the design of the current PDMP models highly affects the operability of the users. The current PDMPs are mainly focused on Schedule II drugs and lack the functionality to address the current opioid crisis, which consists of a wide range of prescription drugs [29]. Additionally, the user interface for the current PDMP is not well developed, consisting of irrelevant and unorganized data with a lack of practical workflow [30]. Apart from that, one of the major limitations in addressing the opioid crisis appropriately is the lack of training provided to prescribers regarding PDMP use [31,32]. Physicians are often unaware of the compliance of their PDMP use, which is further exacerbated by the complexity of the use of such software [29]. Lack of training is often followed by inappropriate data interpretation for a particular patient record, which often leads to incorrect data reporting and loss of valuable time and resources [33-36].

Integrating PDMP into EHR and Use of Data Analytics

Presently, the PDMP is not integrated into the electronic health record (EHR) system, which means that physicians have to access the PDMP separately while logging in to their EHR account [29]. This often leads to a great amount of time wasted. However, physicians compensate for it by putting their prescription details in patients’ charts or often referring back to the charting history to check for the previous prescription drug use. This leads to the lack of appropriate reporting of opioid drugs, causing the ineffective use of PDMP. Considering these factors, it is important to integrate the PDMP into the EHR system, which will bridge the gap, allowing for more effective outcomes [29]. A recent study showed that while there were barriers to the use of PDMP, including system design, administrative burden, lack of training, and lack of perceived usefulness of the PDMP data, there were also several facilitators to the use of PDMP, which included ease of use, appropriate training, and inclusion of complete or detailed data, such as interstate data sharing, data standardization, and an increase in the number of scheduled drug types in data [37]. The use of the PDMP system, whether integrated into the EHR system or not, can be used to assess the trends of prescription drug use for a specific disease condition. This assessment can be performed by using a complex data analytics approach to interpret the data [38]. The data analytics tools will utilize data visualization to minimize the physician’s guesswork based on the diagnosis and the common prescription drugs associated with it.

Discussion

There still remains a great controversy regarding the effectiveness of PDMP in reducing the economic burden and opioid overdoses. It can be perceived that the PDMP system can be beneficial for law enforcement agencies, health regulators, and the overall improvement of public health [21]. However, there are still great barriers to the appropriate utilization of PDMP in the healthcare industry which require attention, along with appropriate federal laws that would enforce prescribers to make effective use of PDMP.

Prescription drug addiction is a multi-faceted problem with varying underlying aspects to it in order to handle the crisis. It is extremely vital to reduce opioid drug abuse while successfully handling pain management among patients to avoid compromising patient satisfaction. The resolution to these issues lies in strengthening and updating the existing PDMP system for prescribers, improving the overall usability for appropriate reporting.

One of the resolutions to overcome the barrier is to integrate the PDMP into the EHR system. One of the first states to integrate PDMP into the EHR system, Indiana, noted a significant difference in the prescribing patterns of the physicians. It was reported that approximately 58% of physicians tended to effectively use PDMP and prescribed less quantities of opioids [29]. This integration can also save a lot of time for physicians, e.g., in an emergency department (ED) setting, further potentially sending out alerts if the patient's prescription history seems to be skeptical.

Additionally, one of the problems reported by the PDMP users is often the lack of real-time data provision [39]. While there is no straightforward solution, it requires updating the existing PDMP system to support the real-time program, which will ease the data reporting and further allow the data interpretation to be less complicated. The improvements in data transmission require updating all systems from the physicians’ end to the pharmacist's end, where the prescription is dispensed [40].

However, real-time access to PDMP by health professionals can greatly contribute to the improved quality of care and adopting a patient-centered approach. The cumulative line of evidence shows that PDMP can assist professionals in sorting out patients with a high risk of prescription drug overdose, which can create an opportunity to develop an early intervention strategy to prevent aggravation of such behavior [21]. This can potentially lead to improved patient-physician relationships, which, in turn, would increase patient satisfaction. 

Regardless of the limitations and barriers, multiple studies have reported a significant correlation between the appropriate utilization of PDMP and a reduced rate of opioid abuse [41,42]. Studies have also shown that the increasing use of PDMP has resulted in reduced prescription drug use while providing more opportunities for governments to regulate the distribution of such drugs and to monitor any form of inappropriate activity [29]. A recent report suggested that the states without PDMP observed an increase in opioid drug use from 2003 to 2009 at a rate of 1.9% per quarter every year, while the states with PDMP implementation observed an increase of only 0.2% [43].

The present study provides evidence that the use of PDMP may potentially decrease prescription drug overdoses in the rural West Virginia population and the barriers that need to be overcome. However, there are several limitations to the present study. There is a gap in the literature regarding the causal relationship between the use of PDMP and deaths by drug overdose, where more research is needed to evaluate the success of the PDMP system. While there are studies providing surveys from a smaller population of healthcare providers with regards to the use of PDMP, a large-scale nationwide survey-based study is needed to evaluate the barriers and improve PDMP use by using the data obtained directly from PDMP users, such as healthcare providers, pharmacists, and nurses. Another potential limitation is that the studies in the literature were primarily based on the US population. While this can be used for the West Virginia population, the effectiveness of PDMP cannot be assessed for the general population worldwide.

Future perspectives

There is an increasing need for the effective implementation of PDMP to avoid aberrant behaviors of opioid addiction among the US population, including West Virginia. Previous studies suggest that opioid addiction can be minimized by assessing the genetic polymorphism to determine the vulnerability of a particular patient [44]. This approach will identify patients with high risks, which will allow for the implementation of targeted prevention strategies and appropriate pain management strategies. Another potential future direction includes assessing the psychology and behaviors of the physician himself. Physicians have a great role in the pain management process. Therefore, it is important to assess the clinical experience of the physician and observe the trends and behavior in order to note clinician-specific variations in prescribing practices. Furthermore, it would be interesting to see an interventional study to see the effectiveness of the proposed measures in this article.

## Conclusions

The primary purpose of this literature review article was to examine the utilization of the PDMP model in the US healthcare industry and the barriers associated with it. The effective implementation of the PDMP system in the West Virginian healthcare industry can reduce the overall burden of prescription drug overdoses and the following economic burden that affects the population. It is important to assess the limitations of the PDMP system and to overcome these barriers. This can be achieved by examining the PDMP system in each state and the strategies that have already been implemented to overcome these limitations. These strategies can be further utilized in West Virginia along with the present data to assess the trends of opioid addiction. Furthermore, there is a constant need for continued improvement in the technological aspect of the PDMP system and establish standards for the use of such a system. There is a need for balance in best medical practice in the West Virginia healthcare industry, which includes finding a reasonable balance between the benefits and addressing the adverse effects of prescription drugs in the process of pain management. This balance can be achieved by the utilization and implementation of PDMP.
